# Retracted: 14‐3‐3ζ promotes gliomas cells invasion by regulating Snail through the PI3K/AKT signaling

**DOI:** 10.1002/cam4.6192

**Published:** 2023-07-13

## Abstract

Li, J, Xu, H, Wang, Q, Wang, S, Xiong, N. 14‐3‐3ζ promotes gliomas cells invasion by regulating Snail through the PI3K/AKT signaling. *Cancer Med*. 2019; 8: 783–794. doi:10.1002/cam4.1950

The above article, published online on 18 January 2019 in Wiley Online Library (wileyonlinelibrary.com), has been retracted by agreement between the journal's editor in chief, Dr Stephen Tait, and John Wiley & Sons Ltd. The retraction has been agreed due to concerns regarding image duplication. A detailed investigation confirmed substantial inconsistencies and errors. As a result, the journal no longer has confidence in the results and conclusions reported.

Li, J, Xu, H, Wang, Q, Wang, S, Xiong, N. 14‐3‐3ζ promotes gliomas cells invasion by regulating Snail through the PI3K/AKT signaling. *Cancer Med*. 2019; 8: 783–794. doi:10.1002/cam4.1950

The above article, published online on 18 January 2019 in Wiley Online Library (wileyonlinelibrary.com), has been retracted by agreement between the journal's editor in chief, Dr Stephen Tait, and John Wiley & Sons Ltd. The retraction has been agreed due to concerns regarding image duplication. A detailed investigation confirmed substantial inconsistencies and errors. As a result, the journal no longer has confidence in the results and conclusions reported.

